# Mechanisms Underlying Type 2 Diabetes Remission After Metabolic Surgery

**DOI:** 10.3389/fendo.2019.00641

**Published:** 2019-09-19

**Authors:** Belén Pérez-Pevida, Javier Escalada, Alexander D. Miras, Gema Frühbeck

**Affiliations:** ^1^Section of Investigative Medicine, Division of Diabetes, Endocrinology and Metabolism, Imperial College London, Hammersmith Campus, London, United Kingdom; ^2^Department of Endocrinology and Nutrition, Clínica Universidad de Navarra, Pamplona, Spain; ^3^Biomedical Research Networking Center for Physiopathology of Obesity and Nutrition (CIBEROBN), ISCIII, Pamplona, Spain; ^4^Obesity and Adipobiology Group, Instituto de Investigación Sanitaria de Navarra (IdiSNA), Pamplona, Spain

**Keywords:** type 2 diabetes, bariatric surgery, insulin resistance, beta-cell function, glucose absorption, glucose utilization, intestinal gluconeogenesis, hepato-portal glucose sensing

## Abstract

Type 2 diabetes prevalence is increasing dramatically worldwide. Metabolic surgery is the most effective treatment for selected patients with diabetes and/or obesity. When compared to intensive medical therapy and lifestyle intervention, metabolic surgery has shown superiority in achieving glycemic improvement, reducing number of medications and cardiovascular risk factors, which translates in long-term benefits on cardiovascular morbidity and mortality. The mechanisms underlying diabetes improvement after metabolic surgery have not yet been clearly understood but englobe a complex interaction among improvements in beta cell function and insulin secretion, insulin sensitivity, intestinal gluconeogenesis, changes in glucose utilization, and absorption by the gut and changes in the secretory pattern and morphology of adipose tissue. These are achieved through different mediators which include an enhancement in gut hormones release, especially, glucagon-like peptide 1, changes in bile acids circulation, gut microbiome, and glucose transporters expression. Therefore, this review aims to provide a comprehensive appraisal of what is known so far to better understand the mechanisms through which metabolic surgery improves glycemic control facilitating future research in the field.

## Introduction

Obesity has become in the last decades the most prevalent metabolic alteration. The pathogenesis of obesity is related to multiple biological processes (genetic, neurobiological, hormonal), being frequently accompanied by psychopathological characteristics (1, 2). Good evidence from meta-analyses and nonrandomized and randomized clinical trials (RCT) has shown that obesity-metabolic surgery is the most effective treatment for patients with type 2 diabetes mellitus (T2DM) (3–12). When compared to intensive medical therapy and lifestyle intervention, metabolic surgery has shown superiority in achieving glycemic improvement, reducing number of medications and cardio-metabolic risk factors, which translates in long-term benefits on cardiovascular morbidity and mortality (3–5, 7, 9–12). Two of these RCTs, extending to 5 years follow-up, have shown that metabolic surgery induces euglycemia in 31–77% of cases (7, 10). Despite the fact that glycemic remission rates differ according to the type of surgery, duration of disease and criteria used to define remission, it has been consistently shown that over 80% of patients maintain good postoperative glycemic control despite reduced or no glucose-lowering drugs (7, 10).

There are different types of metabolic surgery which include the laparoscopic adjustable gastric band, the vertical sleeve gastrectomy (VSG), the Roux-en-Y gastric bypass (RYGB), and the biliopancreatic diversion (BPD) procedure, among other variants. The most common ones performed worldwide are the VSG, RYGB, and the gastric band. The mechanisms underlying glycemic improvement after these procedures have not yet been fully understood but involve a complex interaction among improvements in beta cell function and insulin secretion, insulin sensitivity, intestinal gluconeogenesis, and changes in glucose utilization and absorption by the gut alongside changes in the secretory pattern and morphology of adipose tissue. Therefore, the present review aims to provide a comprehensive analysis of what is known so far to better understand the mechanisms through which metabolic surgery improves glycemic control, in order to facilitate future research in the field.

## Beta cell function and insulin secretion

The physiological ß-cell response is characterized by a biphasic pattern, with an acute initial peak, representing the first phase insulin secretion, which typically happens within the first 30 min after meal consumption. This is followed by a gradually increasing insulin secretion which draws a smaller hump: second phase, 30–180 min after the oral glucose load (13–15). Although plasma glucose concentration is the major stimulus of insulin secretion in the fasting state, gastrointestinal tract-derived signals, mainly the gut hormones released from the endocrine cells, play an important postprandial role. This is explained by the known “incretin effect,” where an enhanced insulin secretion can be observed when a glucose load is given orally as compared to intravenously. This incretin effect can contribute to as much as half of the insulin secretion after a meal (16). This gut-dependent nutrient-induced insulin secretion is mainly driven by two incretin gut hormones: glucagon-like peptide 1 (GLP-1) and glucose-dependent insulinotropic polypeptide (GIP) (16, 17).

The current understanding of T2DM is based on a concept of a gradual failure of pancreatic β-cell function in the context of increasing insulin resistance. Once the pancreas is not able to compensate for this insulin resistance, hyperglycemia ensues and the deterioration of the residual ß-cell reserve is accelerated. This β-cell dysfunction is characterized by the loss of sensitivity (i.e., the slope of the insulin secretion/plasma glucose dose-response relationship or the ability to acutely increase insulin release with increasing glycaemia) and an impaired insulin secretion (i.e., total insulin output in response to a nutrient stimulus) (18, 19).

**Figure 1:**
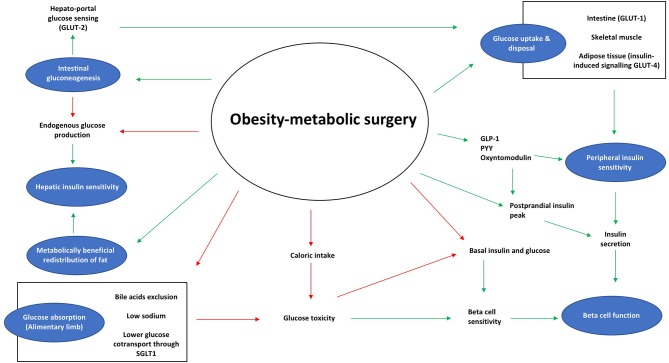
Effects of metabolic surgery on glucose homeostasis. Diabetes remission results from improvements in β-cell function, insulin sensitivity, and changes within the adipose tissue and the gut. Red arrows, represent an inhibitory effect; Green arrows, represent a stimulating effect.

Metabolic surgery (Figure 1) partly restores the dysfunction of the β-cell (19–21). The acute insulin response a surrogate of β-cell sensitivity increases after RYGB, BPD, and VSG or gastric banding (19, 22). This can be observed when using oral tests [oral glucose and mixed meal tolerance tests (OGTT, MMTT)], where an earlier and enhanced post-prandial increase in insulin concentration can be observed as compared to the pre-operative response (22). However, as there is a concomitant improvement in insulin sensitivity, less insulin is required to maintain euglycemia and, therefore, a decrease in the total area under the curve for insulin is observed after all types of procedures (23). The underlying physiological mechanisms are not yet well understood. Several contributors have been proposed in this regard, such as caloric restriction, the removal of glucose toxicity (which can enhance glucose sensing), the improvement in insulin resistance (which decreases the β-cell workload) or changes in gastrointestinal tract-derived signals (i.e., incretin hormones) (24). The enhanced GLP-1 secretion is believed by many to be an important weight loss-independent factor contributing to the postoperative improvement seen in β-cell function following VSG, RYGB, and BPD (20, 22, 23, 25–29). Indeed, antagonism with exendin-(9-39) (Ex-9) of the GLP-1 receptor results in a blunted insulin response after a meal and higher post-prandial glucose concentrations (30). However, these findings are not universal. Some studies also blocking GLP-1 by Ex-9 administration (humans) or in animal models by developing GLP-1 receptor deficient or knockout mice for instance have found different results. When Ex-9 was administered after RYGB surgery, despite observing a worsening in glycemic control, it did not recapitulate the glucose tolerance observed at baseline (31). This is in agreement with another human study comparing RYGB patients with a group undergoing an intensive lifestyle modification therapy where the glucose tolerance deterioration during Ex-9 infusion was similar in both groups (31). Moreover, in GLP-1 knockout mice following surgery the improvements observed in glycemic control, weight or eating behavior did not differ from those observed in wild-type mice (31). Despite supporting the important effect of GLP-1 on glucose-mediated insulin secretion, these findings point out at the relevance of other factors as responsible for the sustained improvement in glycemic control following metabolic surgery. Therefore, some researchers support calorie restriction as the main responsible factor for the acute improvement in the β-cell function seen after metabolic surgery, which reduces glucose levels and therefore the glucose toxicity (32–34). Undoubtedly, the combination of the GLP-1 release with caloric restriction enhances β-cell function in the early post-operative period. This is further achieved through the beneficial effects of weight loss and euglycemia on the β-cell. Nonetheless, it is worth noting that the most important predictor of the degree of postoperative improvement in β-cell dysfunction is the preoperative pancreatic reserve itself: the more “exhausted” the β-cell is before surgery, the less likely a patient is to achieve glycemic remission postoperatively (20, 21, 28, 35).

## Insulin sensitivity

In physiological conditions, when glucose homeostasis is in equilibrium, hepatic glucose production, and renal glucose clearance are balanced with glucose utilization due to the insulin effect in the different tissues. After a meal, insulin release is produced in order to firstly suppress hepatic glucose production to then enhance glucose uptake into peripheral tissues. Hence insulin sensitivity is a reflection of how a given peripheral insulin concentration accelerates glucose disappearance. Thus, in insulin resistance states, higher levels of plasma glucose are observed with higher insulin levels required to compensate for the hyperglycemia.

The mechanisms underlying the improvements seen in insulin sensitivity differ depending on the timing of assessment: early vs. late postoperative period. Within days, an improvement in glycemic control and insulin sensitivity can be observed. Most of the studies have shown that this early improvement is secondary to an increase in hepatic insulin sensitivity as indicated by a reduction in endogenous glucose production (25, 36). In contrast, peripheral insulin sensitivity, including skeletal muscle and adipose tissue, does not change during the early postoperative period, but improves gradually thereafter, exerting a close correlation with weight loss (25, 37). This is consistently seen after all metabolic surgery procedures (38). The exception is the BPD, after which an improvement in both, hepatic and peripheral insulin sensitivity since early stages and to a greater extent compared to weight-matched controls undergoing other obesity interventions can be observed (19, 39).

The increase in hepatic insulin sensitivity is due to a decrease in liver fat content secondary to an increase in lipolysis, which mobilizes fatty substrates to circulation and forces lipid oxidation (37). Interestingly, this can be also achieved with short-term caloric restriction independently of weight loss (40, 41). Therefore, the improvement in hepatic insulin sensitivity seen early after surgery could be just the result of postoperative calorie restriction. Several studies comparing different surgical procedures (VSG, RYGB) vs. a very low-calorie diet (VLCD) have shown that metabolic surgery does not reduce hepatic insulin sensitivity beyond the improvements achieved with caloric restriction (33, 34, 42, 43). However, none of these studies accounted for surgical stress. For instance, C-reactive protein, considered a marker of inflammation, infection or surgical stress quickly increases after any surgery, reaching maximum levels 2–3 days postoperatively. This also happens after metabolic surgery even after non-complicated procedures (44). So, the fact that metabolic surgery had the same improvement in insulin sensitivity as the VLCD means that metabolic surgery may be adding a small additional benefit as the observed just with calorie restriction.

In the late postoperative period, between 3 and 6 months after surgery, weight loss exerts an important role, and is the main driver of the additional improvement in insulin sensitivity. At this stage, an improvement in peripheral insulin sensitivity, which occurs after substantial weight loss takes place and that correlates with the magnitude of weight loss can be observed (19, 25, 36, 37, 39, 45). For instance, a 30% reduction in body mass index predicts a 50% increase in insulin sensitivity as seen in the European Group for the Study of Insulin Resistance (EGIR) cohort among others (19, 39, 46). As mentioned before, BPD surgery is the exception to this statement as it can rapidly improve both, hepatic and peripheral insulin sensitivity before significant weight loss occurs, although the mechanisms are not yet completely understood (47).

The long-term improvement in insulin sensitivity and body weight achieved after metabolic surgery has been mainly ascribed, in VSG, RYGB, and BPD, to the postprandial increase in anorexigenic gut hormones (i.e., GLP-1, peptide YY, and oxyntomodulin), favoring enhanced satiety to a meal which ultimately leads to reduction in calorie and food intake (48). The exception here is gastric banding, after which no increase in anorexigenic hormones takes place (49, 50). The gastric band appears to enhance satiety through neural mechanisms and the subsequent weight loss is the main mediator contributing to the increase in insulin sensitivity after it (49). The increase in gut hormones secretion has been explained through different mediators depending on the type of surgery. In the case of RYGB or BPD, the bypass of the small bowel, bile acids or changes in the gut microbiome have been postulated as possible mediators (51). It has been shown that bile acids indirectly regulate glucose through the G-protein-coupled bile acid receptor, Gpbar1 (TGR5) receptor expressed on L-cells, causing release of GLP-1 upon binding (51–53). With regards to gut microbiota changes, it is still uncertain whether it is directly related to the improvement in glycemic control following surgery. However, if a fecal transplant from healthy volunteers is performed to individuals with metabolic syndrome, an improvement in insulin sensitivity can be observed which correlates with an increased population of butyrate-producing gut microbiota (54). Moreover, some studies have shown that gut microbiota transplantation from RYGB-treated subjects to non-operated ones, results in weight loss and decreased adiposity (55, 56). Nevertheless, in none of these studies glucose nor insulin tolerance was measured (55, 56). Therefore, more studies are needed in order to elucidate to what extent and by which mechanisms the gut microbiome improves glucose metabolism after metabolic surgery. On the other hand, VSG does not have an effect on bile acids circulation. The increase in gut hormones release seem to be secondary to an increase in gastric emptying, which induces a fast transit of nutrients into the small bowel that stimulates gut hormones secretion (57), although the exact mechanism remains unknown (28).

## Glucose absorption

After metabolic surgery glucose metabolism changes within the gut. A lower glucose absorption has been shown to happen following RYGB and VSG, although the mechanism through which this happens differs. After RYGB, undigested nutrients reach the common channel where they meet bile acids and other digestive secretions enabling nutrient absorption. It has been shown that glucose absorption is blunted in the alimentary limb and increases in the common limb secondary to the altered bile acids traffic (58). Endoluminal glucose is absorbed through the apical sodium glucose cotransporter 1 (SGLT1), which incorporates sodium and glucose into the enterocyte from the luminal side. Therefore, the intestinal absorption of glucose requires the presence of sodium, which is originated from bile and other digestive fluids. Thus, after RYGB, there is a modification in bile acid trafficking that results in the alimentary limb not being exposed to these digestive fluids, without sodium present to be co-transported with glucose. In fact, despite SGLT1 expression or function being preserved, bile acids exclusion itself is sufficient to reduce the intestinal sodium-glucose cotransport in the alimentary limb (58). In this context, bile acids modulate the intestinal trafficking of endogenous sodium by decreasing the endoluminal content of sodium in the alimentary limb (58). This also explains why glucose uptake in the alimentary limb can be restored by giving a sodium-rich solution (59). It can be stated that SGLT-2 inhibitors are for the kidney what metabolic surgery is for SGLT-1 in the gut. In fact, some studies have already shown that in patients with T2DM, the inhibition of SGLT1 results in a reduction in postprandial glucose concentrations and an improvement in glycemic control (60, 61).

With regards to VSG, a lower glucose absorption in the small intestine has been shown (59, 62). Following VSG, a large part of the stomach is removed and therefore, there is a reduction in the leptin- and ghrelin-expressing cells. Ghrelin increases appetite, reduces gastric emptying, regulates energy expenditure and decreases glucose-induced insulin release and whole-body insulin sensitivity (63, 64). Therefore, after VSG surgery a negative correlation has been shown between ghrelin concentrations and insulin sensitivity and secretion (64). Gastric leptin is produced in the stomach and secreted into the small intestine, where it is believed to promote glucose absorption by enhancing the glucose transporter-2 (GLUT2) in the jejunum (62, 65). A recent study showed that after VSG surgery there was a decrease in glucose absorption which was enhanced with the addition of an oral gavage of leptin (62). Therefore, it has recently been postulated that after VSG, leptin depletion is one of the main factors contributing to the improvement in glucose homeostasis, rather than gut adaptation as seen after RYGB (62).

## Glucose uptake and utilization within the gut

Despite similar beneficial metabolic effects of VSG and RYGB surgeries, the changes within the gut differ. It has been shown, that after RYGB surgery there is a morphological adaptation of the alimentary limb characterized by mucosal hyperplasia and hypertrophy. These changes in the intestinal mucosa are triggered by the exposure to undigested nutrients by the alimentary limb mucosa and are not found after VSG surgery (59, 66, 67). There is emerging evidence that this hyperplasia and hypertrophy produces a reprogramming of glucose metabolism and increases the metabolic rate in order to meet the higher energetic demand, which boosts the carbohydrate consumption by the gut (59, 67–70). This higher metabolic rate and increased glucose uptake by the alimentary limb can be demonstrated through the use of [^18^F]-fluoro-2-deoxyglucose positron emission tomography-computed tomography where the remodeled intestine exhibits the second highest glucose consumption after the brain (59, 67, 70). In fact, a positive correlation between the intestinal glucose uptake and glycemic improvement was shown, consistent with an improvement in whole-body glucose disposal (67). This process is characterized by an overexpression of the basolateral glucose transporter-1 (GLUT1), which increases the supply of glucose to the enterocyte in the same way as has been shown to do to proliferative cancer cells in response to hypoxia (59). GLUT1 plays an important role in early intestinal tissue growth and therefore is highly expressed in the fetus to then disappear progressively (71). Interestingly, this increase in the glucose utilization by the gut, secondary to the enhanced intestinal expression of GLUT1 after RYGB, is independent of weight loss or improvements in insulin secretion and sensitivity (67). It is worth noting that there is also an increase in the intestinal glucose uptake in the common limb driven by an enhancement of apical SGLT1 activity operating synergistically with the basolateral GLUT1 in order to meet the higher energy requirements (59, 67). All these findings, place the gut within the group of organs/peripheral tissues responsible for increasing glucose disposal after metabolic surgery (59, 67, 70).

## Intestinal gluconeogenesis and the hepato-portal glucose sensing

The glucose release by the small intestine is triggered by two major gluconeogenesis enzymes: glucose-6-phosphatase (Glc6Pase) and phosphoenolpyruvate carboxykinase (PEPCK) (72, 73). When both enzymes are induced, newly synthesized glucose is released into the portal blood. This is detected by the hepato-portal glucose sensing which ultimately modulates the endogenous glucose production by the liver (74, 75). This system requires the presence of a specific glucose transporter (GLUT-2) and is potentiated by GLP-1, through which the portal sensing of glucose appearance suppresses hepatic gluconeogenesis and modulates whole-body glucose disposal, stimulating the glucose uptake by peripheral tissues (76–78). Moreover, portal sensing of intestinal gluconeogenesis induces a reduction in food intake (74, 79–81). These metabolic effects produced by the hepato-portal nervous system seem to take place through the autonomic nervous system around the portal vein which connects to central hypothalamic nuclei (72, 74, 78).

Along these lines, several studies have shown that after RYGB surgery, there is an increased expression and activity of the PEPCK and Glc6Pase enzymes in the distal jejunum and ileum as compared with gastric banding (72). This translates into an increased glucose release by the gut to the portal blood which suppresses hepatic glucose production and food intake (72). These effects where not observed in weight-matched mice after gastric banding, which suggests that at least some of the metabolic improvements seen after RYGB are independent of calorie restriction or weight loss. On the other hand, when GLUT-2 was downregulated in mice undergoing metabolic surgery, there was an impairment in the hepato-portal sensing, which affected insulin sensitivity and body weight (72). Therefore, an increase in the intestinal gluconeogenesis and the stimulation of the hepato-portal glucose sensor via a GLUT-2-dependent pathway has been postulated as one of the mechanisms through which RYGB improves insulin sensitivity and reduces food intake contributing to the resolution of hyperglycemia. However, human studies are needed to corroborate this hypothesis.

## Adipose tissue

It is well-known that adipose tissue dysfunction and an excess of body fat, specifically its central deposition in the abdominal viscera decreases insulin sensitivity and β-cell function and is an independent risk factor for T2DM and cardiovascular disease (82–85). This is due to the fact that the adipose tissue is an active endocrine and paracrine organ which releases numerous hormones, cytokines, and molecules which not only influence body weight, food intake, and energy homeostasis but also regulate glucose and lipid metabolism (86, 87). There is an increasing number of adipocyte-derived hormones which include leptin, adiponectin, resistin, acylation-stimulating protein, retinol-binding protein-4, and visfatin, among others (86, 87). Whilst the existence of as yet unidentified factors controlling body weight and metabolism should be noted (88), what we do know so far is that, except for adiponectin, circulating concentrations of these hormones are increased in obesity and insulin-resistant states, and decrease after weight-loss (51, 89). With regards to cytokines, excess adiposity is characterized by the promotion of chronic, low-grade inflammation which has been implicated in the development of T2DM (87).

As mentioned before, following metabolic surgery significant weight loss takes place. Whilst it would make sense to lose both fat mass and fat-free mass as seen after conventional dieting, it has been shown that after surgically-induced weight loss, body composition improves with a reduction in body fat percentage alongside a minimal drop in fat-free mass (90). Moreover, not only there is an overall body fat loss, but the visceral and intramuscular depot are also reduced (90, 91). This metabolically beneficial redistribution of fat further contributes to the glucose metabolism improvement seen after surgically-induced weight loss: there is an improvement in hepatic insulin sensitivity mediated by the decreased visceral and total adiposity as well as by the refrained muscle mass loss which boosts glucose uptake by the skeletal muscle (91).

But not only body composition improves, adipose tissue itself experiences several changes which include changes in the secretory profile, adipocyte, morphology, and glucose and lipid metabolism. With regards to the adipokines, there is a reduction in leptin and inflammatory cytokines such as TNF-α and several interleukins and an increase in adiponectin concentrations, which translates in a reduction in several cardio-metabolic risk factors (51, 91–95). Moreover, adiponectin concentrations have been shown to correlate with the degree of T2DM remission, being lower in those sub-optimal responders to metabolic surgery (94).

Adipose-specific glucose disposal is enhanced by insulin. The insulin receptor is a tyrosine kinase which activation causes the translocation from the intracellular storage compartment to the plasma membrane of the insulin-sensitive glucose transporter 4 (GLUT4) (96). The activity of the insulin-stimulated AMP-activated protein kinase (AMPK) and GLUT4 transporter are downregulated in patients with obesity and T2DM with the selective inactivation of its gene impairing insulin-dependent adipose glucose disposal leading to T2DM (86, 97–99). It has been shown that after weight loss the insulin-stimulated kinase activity is restored (100) and there is an improvement in insulin-induced signaling and GLUT4 activity in adipose tissue 1-year post RYGB surgery (101–103). These correlated with plasma adiponectin levels and whole-body insulin sensitivity assessed by the hyperinsulinemic euglycemic clamp (101). Several studies have also shown that adipose cell morphology also changes after metabolic surgery: there is an increase in the lipolysis pathways and adipose cell hyperplasia and a reduction in the size which improves whole-body insulin sensitivity (93, 104, 105). All these results, support the role of the adipose tissue as one of the contributors to the glycemic improvement seen after metabolic surgery.

## Conclusion

Metabolic surgery is the most efficient treatment for inducing diabetes remission in obese patients with T2DM. Diabetes remission results from improvements in β-cell function, insulin sensitivity and changes within the gut and adipose tissue. The early improvement seen in postoperative glycemic control is due to an increase in insulin sensitivity secondary to a reduction in hepatic endogenous glucose production and caloric restriction, and an improvement in beta-cell function secondary to an enhancement in GLP-1 release. The long-term benefits in glycemic control are in part due to changes in gut hormone secretion that promote fat mass loss which improves glucose uptake by peripheral tissues (peripheral insulin sensitivity). On the other hand, the exclusion of the proximal intestinal segment after RYGB surgery, changes the gut physiology, affecting glucose absorption and utilization by the gut which contributes to the achievement of diabetes remission after metabolic surgery. Further in-depth understanding of these mechanisms could be used not only to improve the design and effectiveness of these procedures but also to accelerate the identification of targets for drug development.

## Author Contributions

BP-P and GF were the guarantors of this work and, as such, take full responsibility for the work as a whole and the decision to submit and publish the manuscript. JE and AM contributed to discussions and reviewed the paper, and gave their approval to the final version of the manuscript.

### Conflict of Interest Statement

The authors declare that the research was conducted in the absence of any commercial or financial relationships that could be construed as a potential conflict of interest.
